# Comparative analysis of *Legionella lytica* genome identifies specific metabolic traits and virulence factors

**DOI:** 10.1038/s41598-025-90154-5

**Published:** 2025-02-14

**Authors:** Piotr Koper, Jakub Wysokiński, Kamil Żebracki, Przemysław Decewicz, Łukasz Dziewit, Michał Kalita, Marta Palusińska-Szysz, Andrzej Mazur

**Affiliations:** 1 Department of Genetics and Microbiology, Institute of Biological Sciences, Maria Curie-Skłodowska University, Lublin, Poland; 2 Bioinformatics and Biostatistics Laboratory, Institute of Biological Sciences, Maria Curie-Skłodowska University, Lublin, Poland; 3 Department of Environmental Microbiology and Biotechnology, Institute of Microbiology, Faculty of Biology, University of Warsaw, Warsaw, Poland

**Keywords:** *Legionella*, Comparative genomics, Plasmids, Virulence factors, Metabolic traits, Bioinformatics analysis, Comparative genomics, Pathogens

## Abstract

The complete genome of *Legionella lytica* PCM 2298 was sequenced and analyzed to provide insights into its genomic structure, virulence potential, and evolutionary position within the *Legionella* genus. The genome comprised a 3.2 Mbp chromosome and two plasmids, pLlyPCM2298_1 and pLlyPCM2298_2, contributing to a total genome size of 3.7 Mbp. Functional annotation identified 3,165 coding sequences, including genes associated with known virulence factors such as the major outer membrane protein (MOMP), the macrophage infectivity potentiator (Mip), and a comprehensive set of secretion systems (type II, type IVA, and type IVB Dot/Icm type IV secretion system). Notably, *L. lytica* contributed 383 unique genes to the *Legionella* pangenome, with 232 identified effector proteins, of which 35 were plasmid-encoded. The identification of unique genes, particularly those on plasmids, suggests an evolutionary strategy favoring horizontal gene transfer and niche adaptation. The effector repertoire included proteins with domains characteristic of host interaction strategies, such as ankyrin repeats and protein kinases. Comparative analyses showed that while *L. lytica* shares core virulence traits with other *Legionella* species, it has distinct features that may contribute to its adaptability and pathogenic potential. These findings underscore the genomic diversity within the genus and contribute to a deeper understanding of *Legionella*’s ecological and clinical significance. A custom web application was developed using the R Shiny library, enabling users to interactively explore the expanded *Legionella* pangenome through UpSet plots.

## Introduction

*Legionella* spp. are common environmental bacteria that show the ability to parasitize in phylogenetically distant hosts, i.e. freshwater protozoa and human cells, specifically, lung macrophages. In the human host, bacteria can efficiently replicate within a phagocytic cell by forming a structure called the *Legionella*-containing vacuole (LCV). This sophisticated intracellular compartment provides a replication niche and allows bacteria to avoid phagolysosomal degradation, protect them from intracellular defenses, and maintain proper concentration of essential nutrients. This ability of *Legionella* spp. leads to the development of severe pneumonia called Legionnaires’ disease^1^. Legionellosis results mainly from inhalation of *Legionella*-contaminated aerosols and subsequent bacterial replication within alveolar macrophages^2^.

Approximately half of the 73 *Legionella* species identified to date (LPSN database accessed on November 7th, 2024)^3^ have been associated with human disease; however, the overwhelming majority of clinical infections (~ 90%) are caused by *Legionella pneumophila*^4–6^*.* There is a growing interest in bacteria of the genus *Legionella*, which is directly linked to increasing urbanization and global climate change. Air conditioning systems built in response to the drastic heat waves that affect many regions, even in temperate climates, are common places for *Legionella* spp. It is therefore crucial to better understand the ecology and virulence of bacteria belonging to this genus.

Comparative and functional genomics suggested that the *Legionella*–protozoa interaction has shaped the bacterial genome structure extensively^7,8^. A complex evolutionary scenario involving mobile genetic elements, type IV secretion systems, and horizontal gene transfer (HGT) between *Legionella*, amoebae, and other organisms was developed. Moreover, it was revealed that *Legionella* genomes are highly dynamic and characterized by frequent genetic exchange. The analysis of 80 *Legionella* genomes spanning 58 species revealed that genome sizes range from 2.37 Mbp (*Legionella adelaidensis*) to 4.88 Mbp (*Legionella santicrucis*), with GC content varying between 34.82% (*Legionella busanensis*) and 50.93% (*Legionella geestiana*). The GC content of *Legionella* genomes was inversely correlated with genome size, suggesting a strong involvement of HGT, which has resulted in an increase of AT-rich regions during evolution^9^. Importantly, of the 17,992 orthologous gene clusters identified, 5,832 (32%) were strain-specific, while only 1,008 genes (6%) comprised the core genome, demonstrating the high and diverse coding potential of this group of bacteria^10^.

Previous analyses indicated that the plasticity of *Legionella* genomes is associated with the presence of numerous mobile genetic elements, i.e. plasmids, conjugative elements, prophages, and genomic islands^7^. Plasmids have been found in *L. pneumophila*, *Legionella longbeachae*, *Legionella fallonii*, *Legionella hackeliae*, and *Legionella anisa*^11–13^. Currently, within public databases, plasmids are found in 35% of the complete genomes of *Legionella* spp., with sizes ranging from 13 to 373 kbp, constituting 0.1 to 16% of the total genome size. However, megaplasmids (defined as plasmids with a minimum size of 350 kbp)^14^ are rarely detected. Conjugation abilities have been shown only for a few *L. pneumophila* plasmids^15^. Although analysis of the *Legionella* accessory genome has indicated extensive interspecies and intraspecies gene flow, the full extent to which HGT occurs among the *Legionella* spp. remains largely unknown.

The long-term co-evolution of *Legionella* and amoeba has led to the development of highly sophisticated virulence strategies in these bacteria that allow for temporal and spatial fine-tuning of bacterial–host cell interactions. All sequenced *Legionella* species encode a highly conserved type IVB secretion system (T4SS), known as Dot/Icm (defective for organelle trafficking/intracellular multiplication)^16^. The Dot/Icm secretion system is recognized as the major pathogenicity factor of *Legionella*. The system components are encoded by 25 genes organized in two separate genomic regions with conserved gene order and orientation^17^. The Dot/Icm T4SS is essential for intracellular replication, spans both bacterial membranes, and translocates hundreds of virulence factors, termed effector proteins (effectors), directly into host cells. The effectors perform diverse functions, broadly involved in subverting lysosomal bacterial degradation and acquiring nutrients from the host cell^2^. The number of putative effectors encoded by various *Legionella* species varies widely, ranging from 52 in *L. adelaidensis* to 247 in *Legionella waltersii*^17^. Molecular analyses of *L. pneumophila* have identified more than 300 effector proteins as substrates of the Dot/Icm system, with many experimentally confirmed and assigned specific biochemical activities, targets, or functions during infection^18^. However, the pan-genomic pool of putative *Legionella* T4SS effectors is much larger, comprising the repository of over 18,000 proteins, including eukaryotic-like ones, which were shaped during evolution by interdomain gene transfer^10^. Noteworthy, the effector repertoires of different *Legionella* species were found to be largely non-overlapping, with only a few core effectors shared among all species. Species-specific effectors usually displayed low GC content, suggesting exogenous acquisition from natural protozoan hosts^17^.

Eukaryotic-like proteins and motifs are another hallmark of the *Legionella* genus. More than 200 eukaryotic-like proteins and 137 eukaryotic domains (the widest diversity of eukaryotic-like proteins known to date in bacteria), including a unique class of putative bacterial Rab GTPases, have been detected in *Legionella* genomes, suggesting that the manipulation of host signal transduction, protein turnover, and chromatin modification pathways are fundamental intracellular replication/pathogenicity strategies for *Legionella* spp.^1,6,11^. Additionally, other virulence-related traits in *Legionella*, particularly those involved in oxygen binding, iron storage, and host membrane transport, have been postulated to play significant roles in pathogenic mechanisms^11^.

The environmental strain *Legionella lytica* PCM 2298 was originally isolated as a new genus and species of obligate intracellular bacterial parasite of small free-living amoebae from the *Acanthamoeba*-*Naegleria* group^19^. The bacterium causing fatal infections of amoebae was initially named *Sarcobium lyticum* and reclassified as *L. lytica* based on 16S rRNA gene sequence analysis^20^. The entry of the bacteria into a host occurred by phagocytosis, but they grew directly in the cytoplasm rather than within phagosomes. This parasite was readily distinguished from other types of bacteria previously known to live inside amoeba cells by its host cell lytic activity. Morphologically, it is a Gram-negative, rod-shaped bacterium with tapered ends, and the cells are motile due to a polar tuft of flagella^19^.

Beyond PCM 2298, *L. lytica* has been identified in environmental samples such as hot spring water and other aquatic habitats, underscoring its adaptability to diverse ecological niches^21,22^. However, PCM 2298 remains the most studied strain of this species, with detailed structural studies revealing its unique cellular envelope composition, including a distinctive phospholipid and fatty acid profile that differentiates it from other *Legionella* species^23,24^.

In this study, the genome of *L. lytica* PCM 2298 was sequenced using a hybrid approach combining short- and long-read sequencing technologies. To the best of our knowledge, the presented genome is the first complete genomic sequence of the *L. lytica* species. The obtained high-quality genome was subjected to comprehensive annotation and comparative analysis to explore its metabolic potential and identify putative virulence factors.

## Materials and methods

### Culture conditions, DNA isolation, and sequencing

For genomic DNA isolation, *L. lytica* PCM 2298 was cultured in ACES-buffered yeast extract medium until reaching the mid-log phase. Cells were harvested by centrifugation at 4000×*g* for 10 min at 4 °C. DNA extraction was performed using the DNeasy Blood & Tissue Kit (Qiagen, Germany), with modifications for Gram-negative bacteria, including an enhanced lysis step with 20 mg/ml lysozyme at 37 °C for 30 min. For sequencing, DNA libraries were constructed using the NEBNext Ultra II DNA Library Prep Kit for Illumina and sequenced on the Illumina HiSeq 2500 system to generate 250 bp paired-end reads. Additionally, long reads were obtained using the Oxford Nanopore MinION sequencer with an R9.4 flow cell using 1D^2^ chemistry.

### PFGE analysis

Genomic DNA was subjected to pulsed-field gel electrophoresis (PFGE) using the CHEF-DR III system (Bio-Rad). Electrophoresis was performed in a 0.5 × TAE buffer at 6 V/cm, with pulse times ranging from 1 to 20 s over 20 h at 14 °C. Lambda ladder PFGE markers (New England BioLabs) served as size standards. Gels were stained with ethidium bromide and visualized using the Vilber Lourmat gel documentation system. Band patterns were analyzed using ImageJ software to confirm the presence of specific replicons.

### Genome assembly and annotation

Short reads were quality-checked and trimmed using Fastp^25^ to remove low-quality bases and adapters. Long reads were processed using Porechop to remove residual adapters and filtered to retain reads with a minimum length of 4000 bp, in accordance with standard practices for long-read data preprocessing^26^. The genome assembly utilized both short- and long-read data, co-assembled using Unicycler (v0.4.4) with default settings to achieve a highly accurate hybrid assembly^27^. Gaps and uncertain regions were curated through PCR and subsequent Sanger sequencing of the obtained amplicons. The combined genome coverage was approximately 100×. The quality of the assembled genome was assessed using QUAST (v5.0.2) and completeness and contamination levels were further verified using CheckM (v1.2.2)^28,29^. The contamination level was determined to be 1.87%, while genome completeness was 99.59%. Initial automated annotation was performed using the Prokaryotic Genome Annotation Pipeline (PGAP), and further manual curation of genes related to virulence and metabolism was done using the MAISEN tool for more precise annotation^30,31^. Circular genome maps were generated using the GenoVi tool^32^.

### Functional genomic analysis

Functional annotation of genes into Clusters of Orthologous Genes (COG) categories was performed using the GenoVi tool^32^. To gain deeper insight into the specific functional aspects of the *L. lytica* genome, clusters of genes responsible for secondary metabolite biosynthesis were analyzed using antiSMASH v7.0.1^33^, with core biosynthetic gene clusters (BGCs) compared across 65 *Legionella* genomes via BLASTp (≥ 40% identity, ≥ 70% coverage). The resulting data were visualized as a binary heatmap using the ComplexHeatmap R package, with hierarchical clustering applied to species based on BGCs presence/absence profiles.

Antibiotic resistance genes were identified through the Comprehensive Antibiotic Resistance Database (CARD)^34^. Virulence factors were identified using the VFanalyzer tool, an online interface of the Virulence Factor Database (VFDB) with default settings^35^. Raw VF counts were normalized using Z-scores to highlight deviations from the mean. Heatmaps of VF categories were generated using ComplexHeatmap, with hierarchical clustering applied to identify genome-level VF profile similarities. Custom Python and R scripts were used for additional data processing and visualization.

### Phylogenomics analysis

Phylogenetic relationships were explored using autoMLST with the de novo workflow, which automates the selection of single-copy genes and constructs Maximum-Likelihood species trees from scratch^36^. Phylogenomics analyses were performed using the complete genome of *L. lytica* PCM 2298 alongside 65 representative *Legionella* genomes. Digital DNA-DNA hybridization (dDDH) values were calculated using the TYGS server, employing a threshold of ≤ 70% for species delineation, consistent with established standards^3^.

### Comparative genomics and pangenome analysis

For the comparative genomic analysis, a set of 65 reference genomes of *Legionella* species was downloaded from RefSeq (Sup. Tab, 1). Pangenome analysis was performed using Roary with the *-i* flag set to 70 to assess shared and unique genes across the species^37^. General gene annotations for *Legionella* genomes were derived from the NCBI PGAP^30^, while additional functional annotations and metabolic pathways reconstructions were done using eggNOG mapper v2 and the KEGG database for comparative purposes^38,39^.

To facilitate the visualization of the pangenome, a custom web application was developed using R and the R Shiny library (https://www.R-project.org/, https://CRAN.R-project.org/package=shiny). The app dynamically generates UpSet plots to illustrate the distribution of core and accessory genes across the analyzed genomes. The interactive tool is publicly available at https://koper86.shinyapps.io/roary_pangenome/, allowing users to explore pangenome characteristics in real-time.

### Effector protein prediction

The repertoire of potential T4SS effector proteins in *L. lytica* was predicted using OPT4e, a machine-learning-based tool that employs a Support Vector Machine algorithm. This tool uses statistically selected features, including amino acid composition, sequence motifs, and structural properties, to optimize the identification of effector proteins^40^.

To further characterize the predicted effector proteins, Pfam domain annotations were extracted from the functional annotations generated by eggNOG-mapper^39^.

## Results

### Genome structure of *L. lytica*

**Fig. 1:**
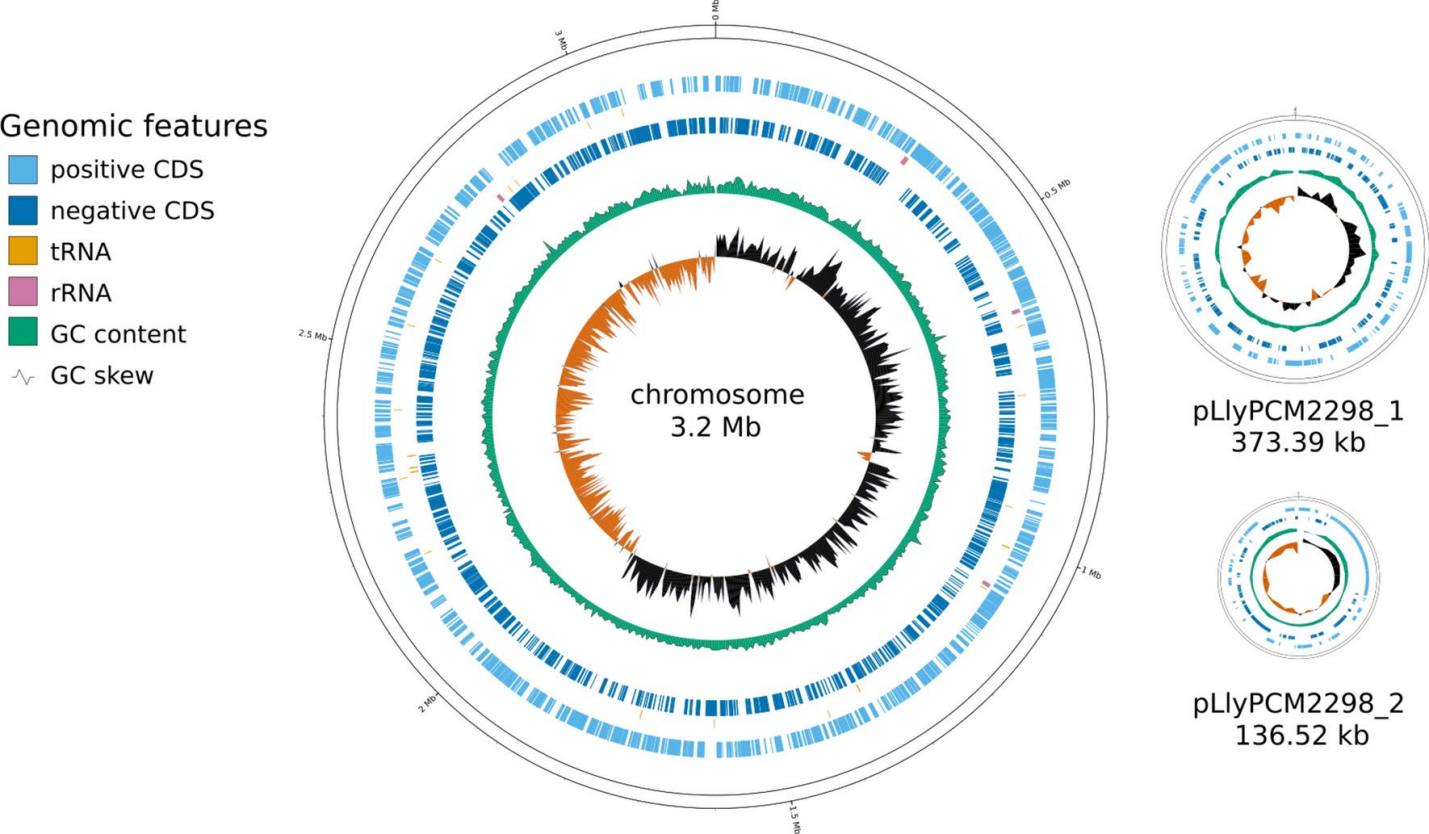
Map of the *L. lytica* PCM 2298 genome, consisting of a circular chromosome (3.2 Mbp) and two plasmids (373 kbp and 136 kbp). The individual rings represent (from outside to inside): genes on the leading strand, genes on the lagging strand, genes for non-coding RNAs, %GC plot, and GC-skew plot.

The genome of *L. lytica* PCM 2298 was sequenced de novo using a combination of short- and long-read sequencing technologies. The total genome size of *L. lytica* is 3.7 Mbp, with a GC content of 39.5%. The genome consists of a single chromosome of 3,198,081 bp and two plasmids, named pLlyPCM2298_1 (hereafter referred to as p1) and pLlyPCM2298_2 (hereafter referred to as p2), with sizes of 373,394 bp and 136,522 bp, respectively (Fig. 1). The genome structure of *L. lytica* is consistent with the replicon profiles previously determined by the PFGE separation (Sup. Figure 1—PFGE).

The total number of determined coding sequences (CDSs) was 3165, with 2743 located on the chromosome, 292 on plasmid p1, and 130 on plasmid p2. This corresponds to a gene density of 857 CDSs per 1 Mbp for the chromosome, and 778 and 955 CDSs per 1 Mbp for plasmids p1 and p2, respectively. Of all predicted CDSs, 2,393 were functionally annotated, giving a functional annotation level of 75%. To further improve this value, selected CDSs were manually curated using the MAISEN tool resulting in a final functional annotation level of 80%. Additionally, 12 rRNAs (4 complete chromosomal 16S–5S–23S rRNA clusters), 43 tRNA genes, and 3 ncRNA genes (RNAse P M1 RNA, 6S RNA, and SRP RNA) were identified.

**Fig. 2:**
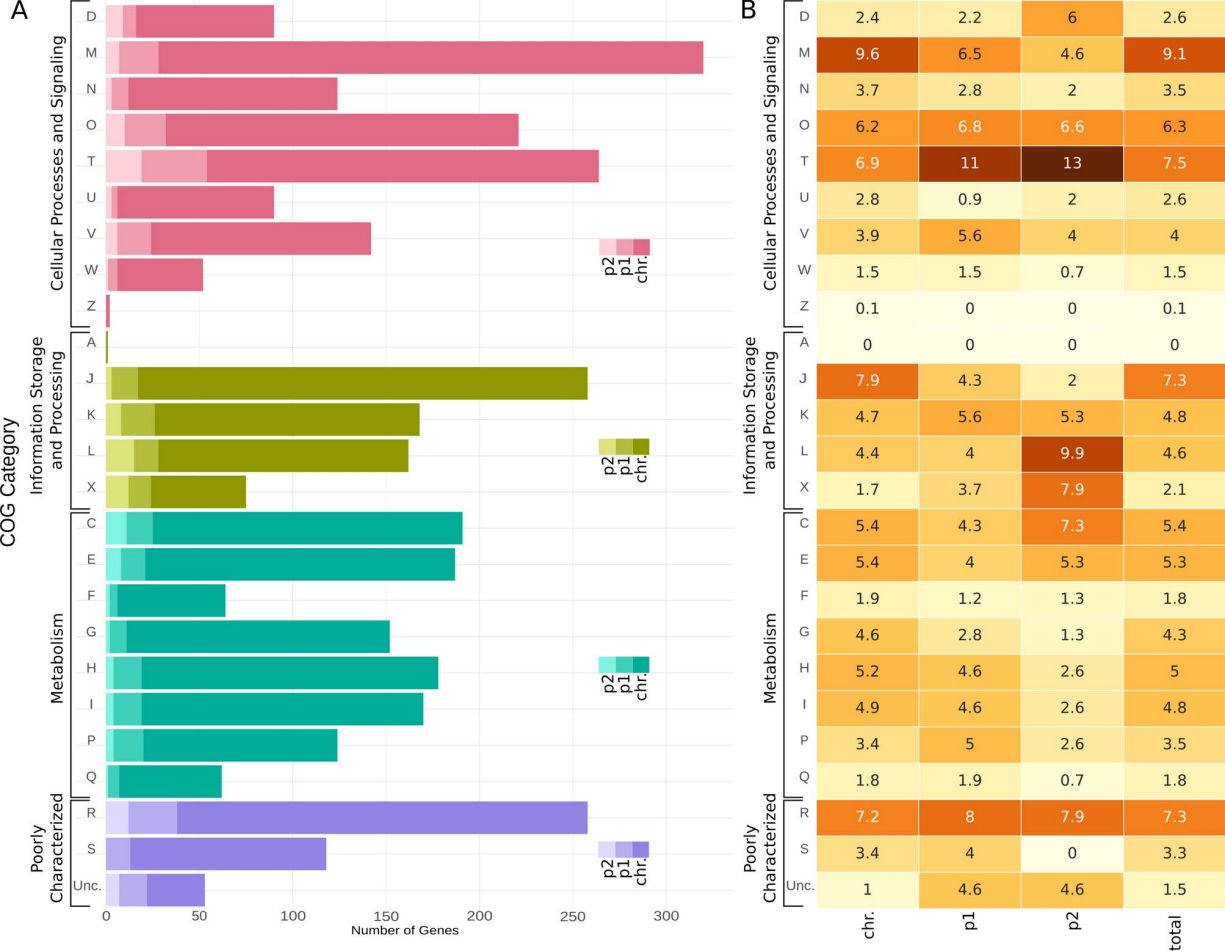
Comparative distribution and frequency of *L. lytica* PCM 2298 genes across COG categories. (**A**) The horizontal stacked bar plot represents the number of genes categorized into COG functions for the three replicons: the chromosome and plasmids p1 and p2. The stacked bars indicate the contribution of each replicon to the total number of genes assigned to each COG category. The colors within each bar distinguish the chromosome (darkest shade), plasmid p1, and plasmid p2 (lightest shade). 'Unc.' denotes genes that were not classified into any COG category. The COG categories are grouped into: Cellular processes and signaling: D (Cell cycle control, cell division, chromosome partitioning), M (Cell wall/membrane/envelope biogenesis), N (Cell motility), O (Posttranslational modification, protein turnover, chaperones), T (Signal transduction mechanisms), U (Intracellular trafficking, secretion, and vesicular transport), V (Defense mechanisms), Z (Cytoskeleton); Information storage and processing: J (Translation, ribosomal structure, and biogenesis), K (Transcription), L (Replication, recombination, and repair); Metabolism: C (Energy production and conversion), E (Amino acid transport and metabolism), F (Nucleotide transport and metabolism), G (Carbohydrate transport and metabolism), H (Coenzyme transport and metabolism), I (Lipid transport and metabolism), P (Inorganic ion transport and metabolism), Q (Secondary metabolite biosynthesis, transport, and catabolism); Poorly characterized: R (General function prediction only), S (Function unknown). (**B**) The heatmap represents the percentage of genes assigned to each COG category for the chromosome, plasmid p1, and plasmid p2. The intensity of color correlates with the relative frequency of genes within each COG category, with darker shades indicating a higher percentage.

The predicted genes of *L. lytica* were functionally classified into COG groups. Interestingly, the highest number of genes (320) were classified into the COG M category (cell wall/membrane/envelope biogenesis). Amongst other COGs overrepresented in the genome were COG T (signal transduction mechanisms) with 264 genes, COG J (translation, ribosomal structure, and biogenesis; 258 genes) and categories comprising unknown genes or with general function prediction (COG S and R, respectively; 376 genes in total) (Fig. 2A).

The distribution of functional genes (COGs) in plasmid p1 was generally similar to that of the chromosome. The main differences were a higher percentage of genes classified in COG T (10.8% for p1 vs. 6.9% for the chromosome) and a lower content of genes in COG M (6.5% for p1 vs. 9.6% for chromosome), as well as those related to translation and ribosome synthesis (COG J). Interestingly, in plasmid p2, 46 out of 151 genes were associated with DNA recombination and horizontal gene transfer (9.9% in COG L and 7.9% in COG X, respectively), and signal transduction (12.6% in COG T) (Fig. 2B).

The plasmids of *L. lytica* encode genes related to carbohydrate and coenzyme metabolism (COG G and H groups, respectively), with lower absolute numbers and percentages compared to the chromosome. Additionally, genes from poorly characterized COG groups were identified on the plasmids, with some percentages exceeding those found in the chromosome.

### *L. lytica* is a distinct genomospecies

Historically, the phylogenetic classification of *Legionella* species has relied heavily on single-gene analyses, particularly of the 16S rRNA gene^17,20^. In the case of *L. lytica*, initial classification positioned the species near the *Legionella*-like amoebal pathogens (LLAP), a result further supported by the bacterium’s poor growth on BCYE medium^19,20,41^.

**Fig. 3:**
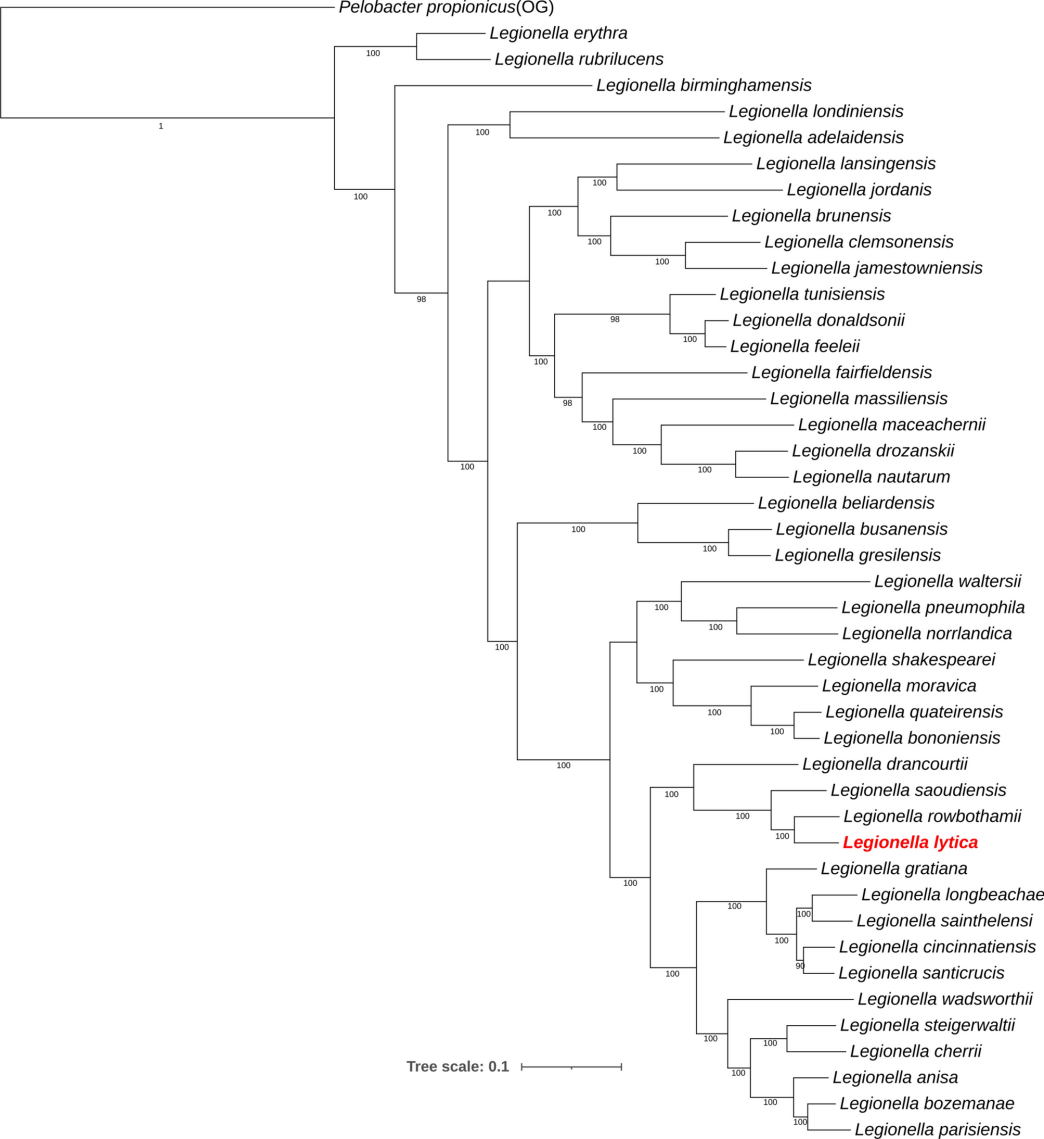
Maximum-likelihood phylogenomic tree based on 84 concatenated core genes, constructed using the autoMLST tool. Fifty reference genomes most closely related to *L. lytica* PCM 2298 were selected based on MASH ANI analysis from a set of 65 genomes analyzed in this study. *Pelobacter propionicus* was used as an outgroup (OG) to contextualize the diversity within *Legionella* species. Bootstrap values greater than 70 are indicated at the nodes. The bar represents sequence divergence.

In this study, we expanded upon previous work by analyzing the complete genome sequence of *L*. *lytica*, enabling a more comprehensive phylogenomic approach. Using the complete genome, we re-evaluated the phylogenetic placement of *L. lytica* within the *Legionella* genus by comparing it against the complete genome sequences of 65 reference *Legionella* strains. In addition, digital DNA-DNA hybridization (dDDH) calculations were performed using the TYGS platform to assess genomic similarity. *L. lytica* showed the highest dDDH values with *L. rowbothamii* LLAP6 (51.3%) and *L. saoudiensis* LH-SWC (42.2%). Based on established thresholds for overall genome relatedness indices (OGRI), with a cutoff of ≤ 70% for species delineation, *L. lytica* is confirmed as a distinct genomospecies. The phylogenetic tree generated by autoMLST corroborated this finding, placing *L. lytica* PCM 2298 on the same node as *L. rowbothamii* LLAP6 and *L. saoudiensis* LH-SWC (Fig. 3).

### *L. lytica* enriches the *Legionella* genus pangenome with more than 380 genes

The pangenome of the *Legionella* genus was constructed using 65 reference genomes of *Legionella* spp., along with *L. lytica*. This analysis revealed a pangenome consisting of 63,051 genes, with 244 classified as core genes (99% ≤ strains ≤ 100%) and 361 as soft core genes (95% ≤ strains < 99%). The majority of genes were categorized as either shell genes (2,930 genes; 15% ≤ strains < 95%) or cloud genes (59,516 genes; 0% ≤ strains < 15%), indicating substantial accessory genome diversity within the *Legionella* genus (Sup. Tab. 2). *L. lytica* contributed 383 unique genes, further enriching the *Legionella* pangenome’s genetic diversity (Sup. Tab. 3).

A custom web application was developed using the R Shiny library to visualize this expanded pangenome, allowing users to interactively explore core and accessory gene profiles through UpSet plots. The tool, available at https://koper86.shinyapps.io/roary_pangenome/, provides a platform for real-time exploration of pangenome characteristics.

### Functional annotation reveals specific metabolic traits encoded in *L. lytica* genome

To gain a deeper understanding of the coding and metabolic potential, the *Legionella* pangenome was annotated against the KEGG database. While the pangenome analysis based on protein similarity identified numerous unique genes, further annotation using KEGG pathways revealed only a limited number of functions that were specific to *L. lytica*. Given that KEGG annotation does not encompass all genes, some functions remain undetected in this analysis. Special focus was placed on functions that were identified as rare or specific to *L. lytica* within the analyzed dataset. Only six such functions were identified in the *L. lytica* genome (Table 1), with each function’s occurrence across reference genomes listed.

**Table 1:** Low-prevalence functions of *L. lytica.*

Locus tag	Replicon	Putative protein product/function	Reference genome presence (species)
*J2N86_RS13365*	Chromosome	Sirohydrochlorin cobaltochelatase (EC 4.99.1.3)	*L. rowbothamii*, *L. jordanis*, *L. fallonii*
*J2N86_RS04905*	Chromosome	D-alanyl-D-alanine dipeptidase (EC 3.4.13.-)	specific
*J2N86_RS01105*	Chromosome	Squalene synthase (EC 2.5.1.21)	*L. saoudiensis,* *L. rowbothamii,* *L. massiliensis,* *L. fallonii*
*J2N86_RS04065*	Chromosome	Undecaprenyl-diphosphatase BcrC (EC 3.6.1.27)	*L. nautarum,* *L. massiliensis,* *L. maceachernii,* *L. drozanskii*
*J2N86_RS14120*	p1	Gamma-tocopherol C-methyltransferase (EC 2.1.1.95)	*L. nautarum,* *L. massiliensis,* *L. fairfieldensis*
*J2N86_RS15310*	p1	Coproporphyrinogen III oxidase (EC 1.3.99.22)	*L. rowbothamii,* *L. gresilensis,* *L. cherrii,* *L. busanensis*

The chromosomal gene *J2N86_RS04905* has been annotated as coding for putative D-alanyl-D-alanine dipeptidase (EC 3.4.13.-), a function uncommon within the *Legionella* genus. According to the BioCyc database, this enzyme plays a critical role in the hydrolysis of the D-alanyl-D-alanine dipeptide, an essential step in peptidoglycan biosynthesis^42^. Although vancomycin resistance mediated by this enzyme is not relevant for Gram-negative bacteria like *L. lytica*, due to their outer membrane providing inherent resistance to glycopeptide antibiotics, studies suggest alternative functional roles. For instance, VanX homologs in Gram-negative bacteria, such as *E. coli*, have been implicated in processes like peptidoglycan recycling during stationary phases or oxidative stress survival. Similarly, we hypothesize that *J2N86_RS04905* may contribute to *L. lytica*’s adaptation to environmental stresses, potentially linked to cellular detoxification or metabolic recycling pathways. Further experimental studies are needed to validate its exact role in *L. lytica*^43^.

The next rare gene, *J2N86_RS01105*, encodes a putative squalene synthase (EC 2.5.1.21), an enzyme involved in the biosynthesis of sesquiterpenoids by catalyzing the conversion of farnesyl pyrophosphate into squalene. This is a remarkable feature, as prokaryotic squalene synthases have been less studied compared to their eukaryotic counterparts. The presence of this enzyme in *L. lytica* contributes to the metabolic diversity observed within the *Legionella* genus and may reflect specific ecological or physiological adaptations^44^.

The gene *J2N86_RS04065*, located on the chromosome, encodes the predicted enzyme undecaprenyl-diphosphatase BcrC (EC 3.6.1.27). This enzyme, similar to the bacitracin resistance protein BcrC of *Bacillus licheniformis*, plays a critical role in bacterial resistance to bacitracin by modulating the cell’s sensitivity and resistance through the regulation of the gene expression. BcrC is involved in glycan biosynthesis and metabolism, particularly in peptidoglycan biosynthesis, by controlling the levels of undecaprenyl phosphate, a carrier lipid essential for bacterial cell wall synthesis^45^.

In addition, two rare genus-wide functions are encoded by the p1 plasmid: putative gamma-tocopherol C-methyltransferase (EC 2.1.1.95) and oxygen-independent coproporphyrinogen III oxidase (EC 1.3.99.22), encoded by genes *J2N86_14145* and *J2N86_15350*, respectively. The first enzyme plays a crucial role in the biosynthesis of vitamin E, specifically facilitating the conversion of gamma-tocopherol to alpha-tocopherol. This step is key in producing the most biologically active form of vitamin E, suggesting that *L. lytica* may utilize this enzyme in metabolic strategies related to antioxidant protection or interaction with the host^46^. The putative oxygen-independent coproporphyrinogen III oxidase catalyzes the conversion of coproporphyrinogen III to protoporphyrinogen IX, a critical step in heme biosynthesis. Heme is an essential cofactor for various biological processes, including oxygen transport and electron transfer. The oxygen-independent nature of this enzyme suggests that *L. lytica* may possess a versatile heme biosynthetic pathway, allowing it to thrive under varying oxygen conditions, potentially contributing to its adaptability and survival in diverse environmental niches or hosts.

### Gene clusters for biosynthesis of secondary metabolites and antibiotic resistance are distributed across different parts of the *L. lytica* genome

Using the antiSMASH tool, several BGCs were identified in the *L. lytica* genome. The most significant, showing the highest degree of similarity to known biosynthesis clusters, were two gene sets located on the chromosome. The first region, named *chr_BGC1* (coordinates 11,842–112,725 bp), contained a trans-AT polyketide synthase, type I polyketide synthase, and non-ribosomal peptide synthetase (NRPS), exhibiting 50% similarity to the legioliulin biosynthetic cluster. Legioliulin is an isocoumarin compound responsible for blue-white autofluorescence in *Legionella* under long-wavelength UV light^47^. The second chromosomal region, *chr_BGC2* (coordinates 1,476,460–1,499,469 bp), encodes a beta-lactone synthetase, with 13% similarity to known fengycin clusters^48^.

Moreover, plasmid-borne BCGs were also found, including: *p1_BGC1* (a putative cyclodipeptide synthase, CDPS, located at 37,243–57,980 bp on p1), *p1_BGC2* (a type III polyketide synthase, T3PKS, at 119,879–161,027 bp on p1), *p1_BGC3* (a type I polyketide synthase, T1PKS, at 163,210–211,972 bp on p1), and *p2_BGC4* (a combined T1PKS/NRPS cluster at 58,917–107,679 bp on p2).

**Fig. 4:**
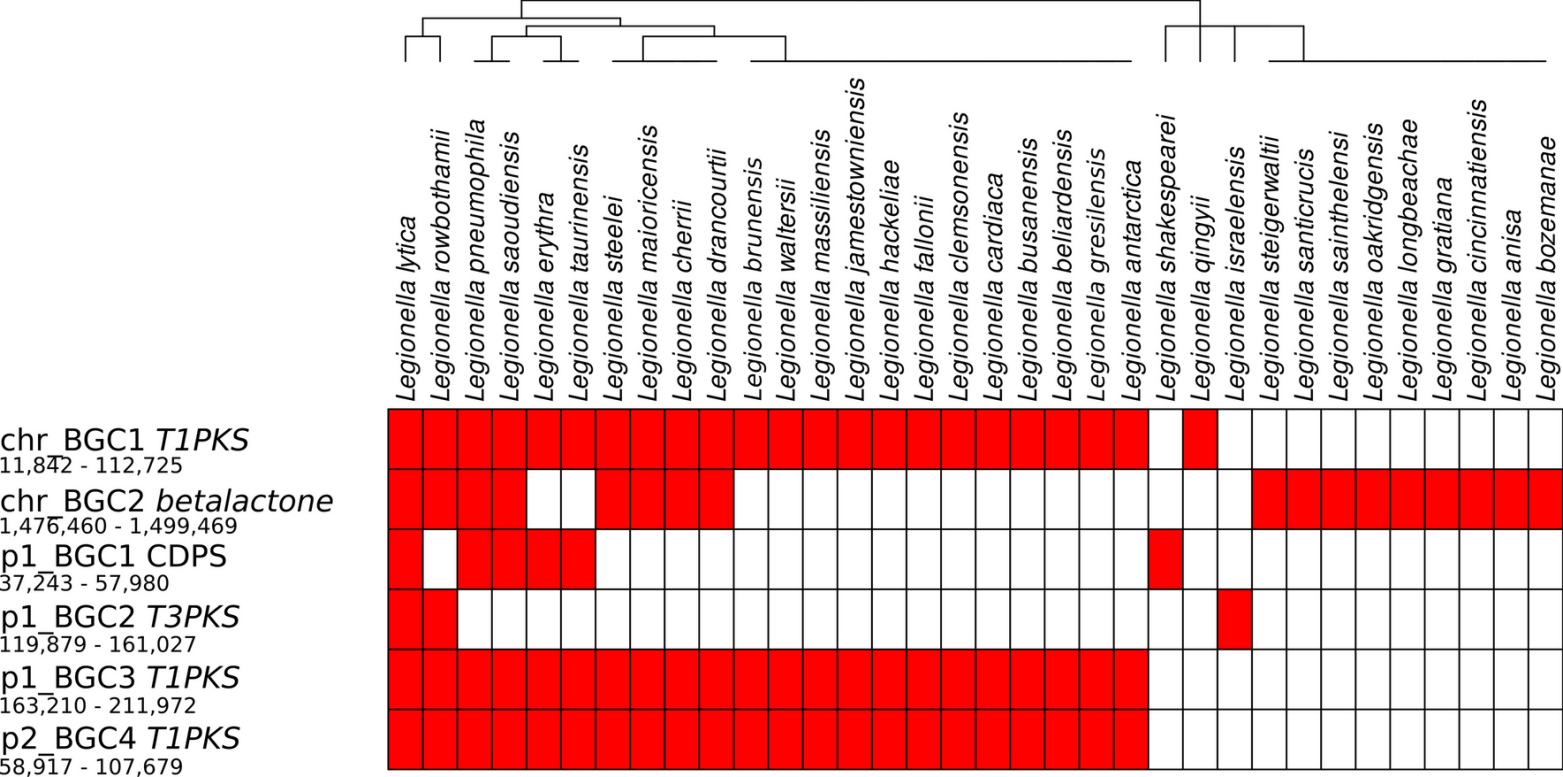
Presence and distribution of biosynthetic gene clusters (BGCs) identified in the *L. lytica* PCM 2298 genome across various *Legionella* species and related genera. The heatmap displays the presence (red) or absence (white) of BGCs, including type I polyketide synthase (T1PKS), beta-lactone, CDPS (cyclic dipeptide synthase), and other polyketide synthase clusters (T3PKS, T1PKS), based on a 70% identity threshold of major genes within each cluster. The genomic locations of these BGCs in the *L. lytica* PCM 2298 genome are shown on the left, along with chromosome and plasmid identifiers and their respective nucleotide positions. The dendrogram above represents the hierarchical clustering of the species based on the similarity of their BGC profiles.

To get deeper inside into these specific gene clusters, a comparative analysis was conducted by performing a BLASTp search of the core biosynthetic gene cluster genes against 65 *Legionella* reference genomes. Hits with at least 40% identity and 70% coverage were considered significant. The occurrence of these BGCs across different species was then examined, and the taxonomy of the species harboring these clusters was analyzed. This approach allowed us to determine the distribution of these core BGCs among *Legionella* species, highlighting both shared and specific biosynthetic capacities, as visualized in Fig. 4. Among the identified BCGs, the chromosomal T1PKS cluster involved in legioliulin biosynthesis, along with the plasmid-borne clusters *p_BGC3* (T1PKS) and *p_BGC4* (T1PKS/NRPS), were the most commonly found across *Legionella* species. Notably, these clusters were consistently observed together, suggesting a potential functional or evolutionary linkage. The co-occurrence of these gene clusters across multiple species underscores their likely importance in the metabolic capacity and adaptation of *Legionella*, particularly in polyketide biosynthesis.

Additionally, a CARD analysis of the *L. lytica* PCM 2298 genome identified two core antibiotic resistance genes, both classified as class A beta-lactamases (locus tags: *J2N86_RS13290* and *J2N86_RS01165*). Importantly, both genes were chromosomally located and implicated in resistance to beta-lactam antibiotics, a broad class that includes penicillins, cephalosporins, and carbapenems. In general, *Legionella* are known for their resistance to beta lactams, and this is a typical feature of this bacterial genus^49^.

### Genes encoding virulence factor categories related to antiphagocytosis, chemotaxis and motility, and toxins biosynthesis are more abundant in *L. lytica*

**Fig. 5:**
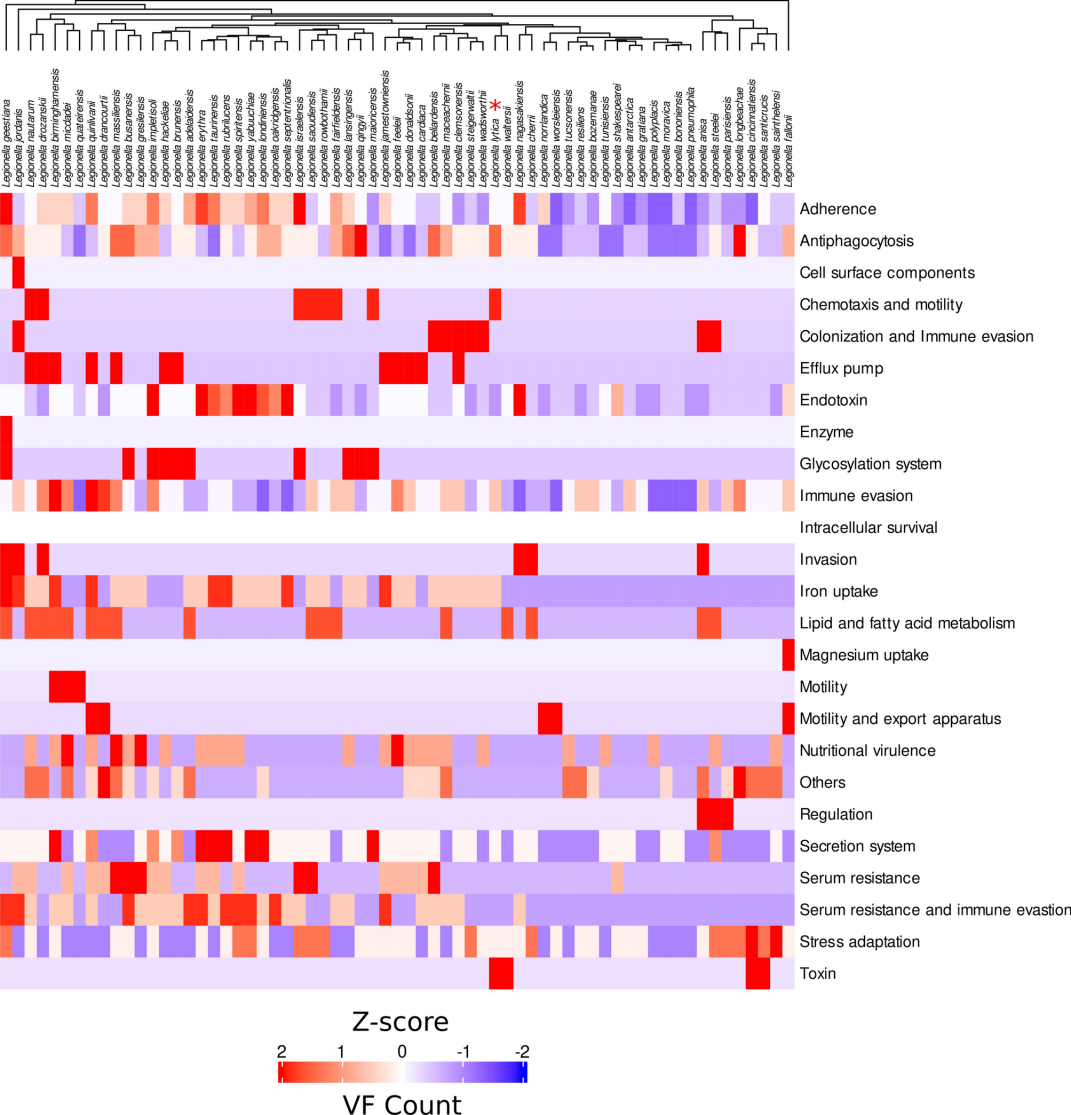
Heatmap of Z-score normalized VF counts across representative *Legionella* genomes. This heatmap illustrates the distribution of VFs across representative genomes of different *Legionella* species, analyzed using the VFDB pipeline. Each row corresponds to a distinct VF class, while each column represents a genome. The Z-scores indicate deviations from the mean VF count for each class, with red indicating higher-than-average VF counts, blue indicating lower-than-average counts, and white representing values near the mean. Genomes are clustered based on their VF profiles, with genus and species names displayed in italics at the top of the heatmap. The VF profile of *L. lytica* is marked with a red asterisk.

The virulence potential of *L. lytica* was assessed by comparing its gene content with the curated and comprehensive database of bacterial virulence factors (VFDB), containing information about the genes encoding well-characterized virulence factors (VFs). This analysis included a comparison of *L. lytica* PCM 2298 with other reference *Legionella* species, providing a detailed examination of VF profiles across the genus. Following the analysis, a heatmap was generated to visualize Z-score normalized counts of recognized VFs within specific VF categories, highlighting differences and similarities in VF content across the reference genomes (Fig. 5).

The comparison clearly shows that *L. lytica* encodes all the relevant and characteristic virulence factors typical for the *Legionella* genus. Diverse surface structures of *L. pneumophila*, such as lipopolysaccharides (LPS), flagella, pili, major outer membrane protein (MOMP), macrophage infectivity potentiator (MIP), and the 60-kDa heat shock protein (Hsp60), have been recognized to play a potential role in pathogenesis^50^. Genes encoding all these elements are present in the *L. lytica* PCM 2298 genome (Sup. Tab. 4).

Notably, *L. lytica* exhibits significant Z-score deviations in several VF categories compared to other *Legionella* species, including antiphagocytosis, chemotaxis and motility, and biosynthesis of toxin. In the antiphagocytosis category, *L. lytica* exhibited a Z-score of 1.5, indicating higher-than-average VF counts (Fig. 5). In this category, the genes *wcbC* (*J2N86_RS13895*) and *wzm* (*J2N86_RS13920*), potentially involved in extracellular capsule synthesis, were found in only a few species, such as *L. longbeachae*, *L. massiliensis*, and *L. qingyii* (Sup. Tab. 4). Capsule structures are critical for bacterial pathogens, providing a physical barrier against phagocytosis by host immune cells, effectively shielding the bacteria from the host’s primary defense mechanisms. Furthermore, the extracellular capsule can interfere with recognition and response by the host immune system, thereby enhancing the bacteria’s ability to persist and proliferate within the host environment^51^. To date, *L. longbeachae* is the only *Legionella* species in which a capsule has been identified^52^.

In addition, *L. lytica* carries the gene *J2N86_RS15225* on plasmid p1, which encodes a putative type III hemolysin, a homolog of the *hlyIII* gene from *Bacillus cereus*^53^, resulting in the high Z-score in the toxin biosynthesis category (Fig. 5). While *Legionella* species are known for hemolytic properties and genes encoding various hemolysins have been found in their genomes, this is the first report of a plasmid-encoded hemolysin gene in the genus ^54,55^.

Particular attention was given to the genes encoding protein secretion systems identified in *Legionella* spp. The genomes of *L. pneumophila* are known to code for various secretion systems, such as the putative type I Lss, type II PilD-dependent Lsp, type IVA Lvh, and type IVB Dot/Icm, while in the case of non-*pneumophila* strains there is much more variation, with individual secretion systems distributed randomly^56^. In the case of *L. lytica* PCM 2298, complete Lsp type II and Lvh type IVA systems were found on the chromosome, as well as both coding regions for the T4BSS Dot/Icm system (Sup. Tab. 4).

### Distinct effectors of *L. lytica* enrich the pathogenic potential of the *Legionella* genus

The analysis of the *L. lytica* genome revealed a complete type IVB secretion system (T4BSS) encoded on the chromosome. The T4BSS is known for its role in facilitating intracellular infection by translocating effector proteins into host cells, a critical aspect of *Legionella* pathogenicity. To further investigate the repertoire of *L. lytica*'s virulence factors, we employed the OPT4e tool.

The OPT4e predictions identified 232 potential effector proteins encoded within the *L. lytica* PCM 2298 genome (Sup. Tab. 5). Notably, 35 of these effectors are plasmid-encoded, with 30 located on plasmid p1 and five on plasmid p2. Interestingly, 63 of the 232 potential effector proteins encoded in the *L. lytica* genome were part of the 383 unique gene sets detected in the *Legionella* pangenome, including 19 effectors encoded on plasmids (Sup. Tab. 3).

Furthermore, our analysis confirmed that *L. lytica* harbors all seven core effectors identified by Burstein et al., which are conserved across the *Legionella* genus and play essential roles in intracellular replication and host manipulation. This conservation underscores the functional relevance of these effectors across *Legionella* species, while the additional distinct effectors identified in *L. lytica* may contribute to species-specific adaptations and pathogenic traits. These findings align with broader observations from genomic studies, which showed that effector repertoires across *Legionella* species are largely non-overlapping, with only a few core effectors shared among species^17^.

**Fig. 6:**
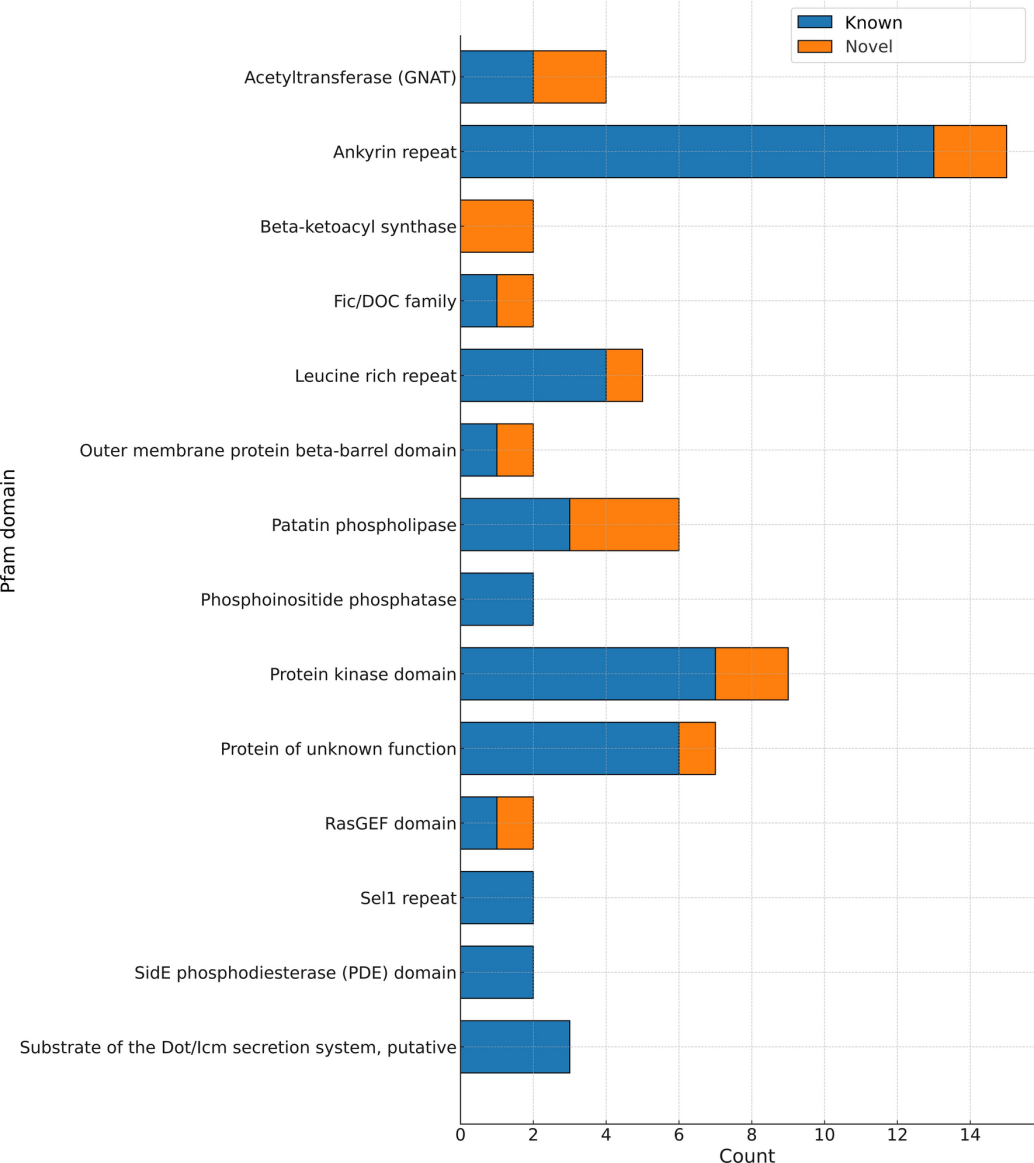
Stacked bar plot of Pfam domains identified within potential effector proteins encoded in the *L. lytica* PCM 2298 genome. The bars represent the counts of each domain, with “Known” in blue and “Novel” domains in orange. Domains are sorted alphabetically, and only those with at least two counts are included. The “Known” category includes predicted effectors that are shared with other genomes in the analyzed dataset, while the “Novel” category represents effectors from the unique portion of the pangenome, not shared with other *Legionella* species*.*

To gain a more comprehensive view, protein domain identification analysis was performed using the Pfam database. This analysis revealed the presence of 56 distinct protein domains within the set of effectors analyzed. The most abundantly represented elements were ankyrin repeats, protein kinase domains, and patatin phospholipase (Fig. 6). Interestingly, many of the identified domains are of eukaryotic origin, which is a characteristic feature of effector proteins in the *Legionella* genus^10^.

## Discussion

### Genomic architecture and adaptive potential

The genome structure of *L. lytica* PCM 2298, with its notably large plasmid p1, represents a unique feature among known *Legionella* genomes. While *L. pneumophila* is extensively studied, insights into non-*pneumophila* species, such as *L. lytica*, are essential for a more comprehensive understanding of *Legionella* genomic diversity and adaptability. The analysis of these non-dominant species helps reveal structural variations that may contribute to niche specialization and environmental adaptability.

The genome of *L. lytica*, with its 3.7 Mb size and 39.5% GC content, falls within the known variability of the *Legionella* genus, which ranges from 2.37 Mb in *L. adelaidensis* to 4.88 Mb in *L. santicrucis*, and GC content spanning from 34.82% in *L. busanensis* to 50.93% in *L. geestiana*. This places *L. lytica* near the midpoint of both genome size and GC content variability, reflecting the diverse genomic architecture seen across the genus^17^.

The COG distribution in *L. lytica*, resembles the one for other *Legionella* genomes and reflects its both host-adapted lifestyle and ecological versatility. According to Hugoson et al., the evolution of *Legionella* genomes, particularly in COG categories such as cell wall/membrane biogenesis and signal transduction, aligns with their need for structural resilience and environmental responsiveness. This is evident in the substantial representation of COGs involved in cellular processes and signaling^57^.

Plasmids in *Legionella* spp. have been shown to carry genes that are essential for adaptation to specific environmental pressures, such as survival in amoebal hosts and resistance to stressors commonly encountered in water systems^58^. The full significance of plasmid content in *Legionella* remains underexplored, as the majority of genomic data available pertains to *L. pneumophila*, with far less known about other species.

Plasmid p1, the largest plasmid identified within the *Legionella* genus according to currently available genomic data, shows a notable enrichment in COG T (signal transduction mechanisms). This indicates that p1 may be involved in regulatory processes that enhance the bacterium’s ability to respond to environmental stimuli. Such functions are crucial for survival in host-associated or variable environments, where rapid adaptation is necessary^59^.

In contrast, plasmid p2, although smaller, carries a higher proportion of genes related to COG L (replication, recombination, and repair) and COG X (mobilome elements, such as transposons). This suggests that p2 plays a pivotal role in genetic plasticity, facilitating horizontal gene transfer and genetic rearrangements. The higher representation of these COG categories highlights p2’s potential function in promoting genomic variability and adaptability. This aligns with the concept that smaller plasmids often act as reservoirs for genes that confer adaptive advantages^60^.

Furthermore, the presence of genes representing poorly characterized COG groups on the plasmids, with some percentages exceeding those found in the chromosome, suggests that plasmids might harbor unique and unexplored functions. These functions could provide adaptive advantages in specific, yet unrecognized, environmental conditions, influencing the bacterium’s phenotypic potential and overall metabolic diversity.

### Phylogenomic position and pangenome

The phylogenetic positioning of *L. lytica* adjacent to *L. rowbothamii* and *L. saoudiensis* within cluster IV, as described in Gomez-Valero et al.'s work, highlights its shared evolutionary trajectory with other non-*pneumophila* species^10^. This cluster represents a lineage characterized by extensive gene acquisition events, potentially linked to ecological and virulence adaptations. Notably, members of this cluster, such as *L. longbeachae*, are capable of causing human infections, albeit in specific contexts. For instance, *L. longbeachae* is frequently associated with Legionnaires’ disease in Australia, where exposure to potted soil serves as a major source of infection^1^. This sugests that environmental niches play a critical role in shaping the virulence potential and transmission pathways of cluster IV species. The relatively larger genome sizes within this lineage, including *L. lytica*, suggest an adaptive strategy driven by the retention and integration of horizontally acquired genes, allowing them to exploit specific ecological niches and develop distinct virulence schemes. This evolutionary balance between intracellular specialization and environmental adaptability highlights the genomic plasticity that underpins the survival strategies of *Legionella* species in diverse ecological and host-associated niches.This observation is further supported by pangenome analysis, which identified 63,051 orthologous gene groups with only 244 core genes and a vast majority classified as shell and cloud genes. This finding is consistent with Burstein et al.'s study, which analyzed 38 *Legionella* species and found 16,416 clusters of orthologs with a core genome comprising only 1,054 genes^17^.

The presence of 59,516 cloud genes in *Legionella* pangenome aligns with the high variability and gene gain observed by Gomez-Valero et al., who documented that more gene acquisition events than losses have driven *Legionella* evolution, which is clearly exemplified by the presence of larger genomes across different *Legionella* phylogenetic clades. This pattern indicates that the *Legionella* genus has evolved to adapt to varied environments through extensive horizontal gene transfer and gene conservation strategies^10^.

Joseph et al. highlighted that in their analysis, 5832 (32%) of the identified orthologous gene clusters were singletons (strain-specific), further corroborating the notion of an open pangenome with significant gene diversity across species^61^. The 383 unique genes contributed by *L. lytica* follow this trend, demonstrating their role in expanding the functional capacity of the genus and enhancing its ecological adaptability.

The KEGG-based functional annotation of *L. lytica* revealed that, despite contributing 383 unique genes to the *Legionella* pangenome, only a limited number of unique functional categories were identified. This aligns with Gomez-Valero et al.'s observations that while *Legionella* species often possess large accessory genomes, their metabolic capabilities can vary significantly, reflecting specific ecological adaptations^10^. Notably, from the unique genes contributed by *L. lytica*, 199 were annotated as hypothetical proteins and 16 contained DUF (domain of unknown function) domains, suggesting that many of these singletons have uncharacterized functions (Sup. Tab. 3). This highlights the challenge of fully understanding the functional implications of such gene contributions, as a substantial portion of the accessory genome remains without clear functional classification.

Among these unique contributions, *J2N86_RS04905*, a putative D-alanyl-D-alanine dipeptidase, is particularly noteworthy. Although its canonical role in vancomycin resistance is irrelevant for Gram-negative bacteria like *L. lytica*, this gene may have an alternative function, potentially in peptidoglycan recycling or cellular stress responses, as suggested by homologous genes in other bacteria. Such processes could provide adaptive advantages under environmental stress or nutrient-limited conditions^43^.

Another rare metabolic feature, *J2N86_RS01105*, encodes a putative squalene synthase, which catalyzes sesquiterpenoid biosynthesis. The presence of this enzyme highlights the metabolic diversity of *L. lytica* and its potential ecological specialization. While prokaryotic squalene synthases are understudied, their role in *L. lytica* may reflect a specific adaptation, possibly related to membrane dynamics or stress resistance^44^.

Plasmid p1 of *L. lytica* also encodes gamma-tocopherol C-methyltransferase, facilitating the production of the biologically active form of vitamin E. This function might support antioxidant protection or host interactions. Another plasmid-encoded gene, oxygen-independent coproporphyrinogen III oxidase, suggests a versatile heme biosynthetic pathway that may enable *L. lytica* to thrive under diverse oxygen conditions, potentially contributing to its ecological adaptability^46^.

### Virulence potential of *L. lytica*

A key aspect of the virulence profile of *L. lytica* is the wide range of protein secretion systems it possesses. The genome analysis revealed the presence of a complete type IVB Dot/Icm secretion system (T4BSS) on the chromosome, along with complete type II (Lsp) and type IVA (Lvh) secretion systems. These systems are essential for interacting with host cells, translocating effector proteins, and modulating host functions to support bacterial survival and replication.

OPT4e analysis identified 232 potential effector proteins in *L. lytica*, with 35 encoded on plasmids—30 on plasmid p1 and five on p2. Importantly, 63 of these effector proteins were among the set of 383 unique genes contributed by *L. lytica* to the *Legionella* pangenome. This supports findings by Gomez-Valero et al., who noted that effector repertoires in *Legionella* are highly diverse, with species-specific sets providing functional differentiation within the genus^10^. Such non-overlapping effector profiles suggest that *L. lytica* has evolved a pathogenic toolkit, that enhances its adaptability to different environmental and host conditions^17^.

The identification of 56 distinct protein domains within *L. lytica* effectors, including ankyrin repeats, protein kinase domains, and patatin phospholipase, reflects its complex interaction strategy with host cells. These domains, many of which are of eukaryotic origin, are known to modulate host cellular processes, further supporting the sophisticated nature of *Legionella*’s pathogenic mechanisms^17^. The presence of eukaryotic-like domains is consistent with the genus-wide adaptation to exploit host cell machinery for survival and replication.

Virulence factor analysis also highlighted the presence of key elements such as MOMP, Mip, and the complete set of genes for capsule synthesis, including *wcbC* and *wzm*. The capsule-associated genes, found in only a subset of *Legionella* species like *L. longbeachae*, suggest enhanced protection against phagocytosis, enabling *L. lytica* to better evade immune responses^52^. Additionally, the plasmid-encoded type III hemolysin gene (*J2N86_RS15225*) could facilitate membrane disruption during host infection, underscoring the contribution of plasmids to the unique virulence arsenal of *L. lytica*^54^.

## Conclusions

The comprehensive analysis of *L. lytica* PCM 2298 reveals a genome equipped with both conserved and unique virulence features, underscoring its adaptability and potential for diverse host interactions. The presence of a complete set of secretion systems and a distinct effector repertoire positions *L. lytica* as a significant contributor to the *Legionella* genus’s genomic and functional diversity. We hypothesize that the identification of unique genes, particularly those on plasmids, suggests an evolutionary strategy favoring horizontal gene transfer and niche adaptation. Additionally, the development of a custom R Shiny web application provides an interactive platform for exploring the pangenome characteristics of *Legionella*, facilitating real-time data visualization and analysis. These insights enrich our understanding of *Legionella* pathogenesis and pave the way for further studies on the ecological roles and infection mechanisms of non-*pneumophila* species.

## Supplementary Information

Supplementary Information 1.

Supplementary Information 2.

Supplementary Information 3.

Supplementary Information 4.

Supplementary Information 5.

Supplementary Information 6.

## Data Availability

The genome assembly of *Legionella lytica* PCM 2298 and associated raw sequencing reads are publicly available under the NCBI BioProject accession number PRJNA708212. The genome assembly can be accessed in GenBank under accession number GCF_023921225.1, and the raw reads are stored in the Sequence Read Archive (SRA) within the same BioProject.
