# Supplementary figures and images for: Comparative analysis of Legionella lytica genome identifies specific metabolic traits and virulence factors
Source: Sci Rep. 2025 Feb 14;15:5554. doi: 10.1038/s41598-025-90154-5 (PMC11828895; doi:10.1038/s41598-025-90154-5)

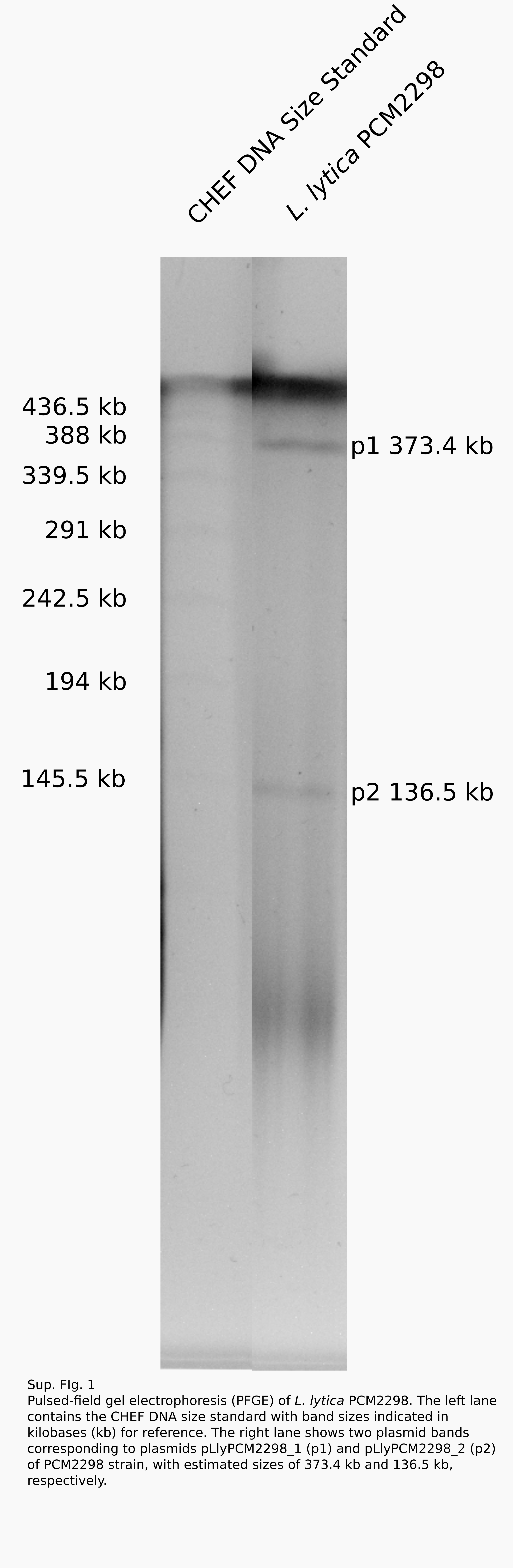

Supplement: Supplementary file 1 — Supplementary Information 1. [file 41598_2025_90154_MOESM1_ESM.png]
